# New Working Environments: Mission Completed?

**DOI:** 10.1007/s00062-021-01020-6

**Published:** 2021-06-17

**Authors:** Jennifer Linn

**Affiliations:** Institute of Diagnostic and Interventional Neuroradiology, University Hospital Carl Gustav Carus Dresden, Dresden, Germany


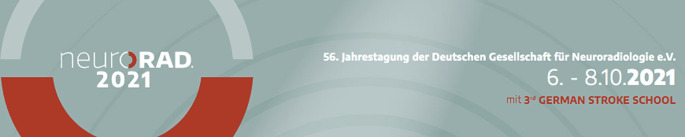


Dear colleagues,

It is my great pleasure to invite you to neuroRAD 2021, the 56th annual meeting of the German Society of Neuroradiology, offered as a comprehensive virtual congress from 6th to 8th October 2021.

Due to the corona pandemic, the motto “New working environments”, which we initially choose for the 2020 congress, became true in a way that no one of us could have anticipated. We all were faced with a new reality both in our private as well as in our working life. Therefore, neuroRAD 2021 resumes this motto by adding a question: “New working environments: mission completed?”. The impact of last yearʼs enormous challenges but also considerable achievements on both the nature and the content of our daily work will be discussed in various formats of our congress and in a key note lecture by Sascha Friesike, Professor of Digital Innovation Design at the Berlin University of the Arts and Director of the Weizenbaum Institute for Networked Society, one of the highlights of our meeting.

NeuroRAD 2021 offers state of the art lectures on recent developments in neuro-oncology, neurodegenerative diseases, interventional neuroradiology and imaging of the spinal canal. In addition, special emphasis of this year’s congress lies on the management of vasospasm and on the pathomechanism and differential diagnosis of autoimmune disorders and cerebral small vessel diseases. We are honored to welcome Prof. Joanna Wardlaw, University of Edinburgh, as a key note speaker on the latter topic.

All accepted scientific abstracts are presented as “power pitches” as part of the abovementioned state of the art and special focus sessions as well as in dedicated scientific rounds.

The *Forum Junge Neuroradiologie* will focus on current developments in education and training and discuss different career opportunities in neuroradiology. Interactive teaching on neuroanatomy, pediatric neuroradiology, spinal diseases as well as infectious and metabolic disorders will be provided by our Fit For Neuroradiology program.

We are glad to announce that neuroRAD 2021 also comprises the 3rd German Stroke School, a dedicated lecture and hands-on simulation course for interventional stroke treatment. For the first time the German Stroke School lecture curriculum, which covers all relevant topics related to interventional stroke treatment, will be free for all neuroRAD 2021 attendees! For a maximum of 32 participants a full day hands-on training using the latest simulation techniques will be available.

Last but not least, neuroRAD 2021 also includes virtual social events to give you the opportunity to meet your friends and colleagues.

Please explore our website www.neurorad.de for further details regarding the congress program, abstract submission and registration for neuroRAD 2021.

I am very much looking forward to seeing you at our (virtual) congress in October 2021!

Yours sincerely,

Prof. Dr. Jennifer Linn, Dresden

